# Immobilized Metal Affinity Beads for Recombinant Protein Analysis by Desorption Electrospray Ionization Mass Spectrometry

**DOI:** 10.1021/jasms.6c00155

**Published:** 2026-07-17

**Authors:** Christopher James Taylor, Sumana Parvin, Fazeleh Shafiepour, Frederick Stull, Andre R. Venter

**Affiliations:** Department of Chemistry, 4175 Western Michigan University, Kalamazoo, Michigan 49008-5413, United States

## Abstract

We address the limitations of the low binding capacity of smooth metal affinity surfaces (below 1.661 femtomoles per mm^2^) and the well-documented low sensitivity of DESI-MS toward larger proteins by increasing the specific surface area of the sampling substrate, thereby providing a greater binding capacity per unit area of the desorption footprint. This was achieved using Ni-NTA-functionalized magnetic beads adhered to a flat surface. Close packing of the spherical beads provides a greater surface area, yielding an increased protein binding capacity as high as 4.028 nmol per mm^2^ of sampled surface. The magnetic properties of these beads allow for simplified workflows and efficient protein recovery directly from cell lysate prior to mass spectrometric analysis. The approach is demonstrated by purifying known concentrations of His-labeled S-adenosylhomocysteine nucleosidase and lactate oxidase. The known enzymatic reactions for each protein are shown directly from the captured proteins adsorbed on the beads by DESI-MS. Acidifying the desorption solvent disrupts interactions between chelated nickel and histidine, allowing for the release of the protein and its analysis by DESI-MS.

## Graphical abstract

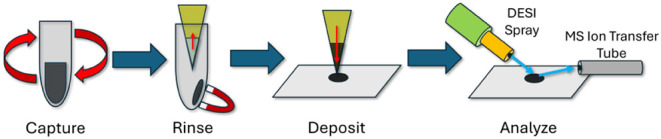

## Introduction

As sequence libraries have increased in size, so too has the number of sequences without a known activity. The downstream processing and recovery of proteins of interest from complex expression matrices is commonly a burdensome undertaking, which prevents high-throughput screening of these sequences. Mass spectrometry is well-positioned to bridge this gap. It is quite common to hyphenate various separation techniques with mass spectrometry, but these methods are often onerous as they require expensive supplemental equipment and extensive sample preparation, which may make deorphanizing proteins problematic.

Techniques in direct and ambient ionization mass spectrometry (AIMS) provide many benefits in the analysis of proteins by mass spectrometry. Desorption electrospray ionization mass spectrometry (DESI-MS) is a specific example of AIMS, a method where sample preparation is minimal and mostly occurs during the analysis.^1^ In DESI-MS, a charged, pneumatically assisted solvent spray is directed at a sample that has been deposited onto a surface. This spray expels analyte from the surface via a “droplet pickup mechanism”.^2,3^ The charged stream of solvent droplets forms a microlocalized liquid layer over the surface sample into which analyte molecules are extracted or dissolved.^4–6^ Continued bombardment of the surface with solvent droplets leads to the ejection of progeny droplets, which now contain analyte. These droplets then undergo electrospray ionization mechanisms to produce detectable ions.^7–9^ DESI-MS does, however, struggle with larger proteins^10^ mostly due to slow dissolution, adduct formation,^10^ and aggregation during analysis with denaturing desorption solvents.^11^ Ion mobility,^12^ changes in configuration,^13^ liquid sample DESI,^14^ the addition of ammonium bicarbonate,^15^ serine,^16^ solvent vapors,^17^ and other approaches have been used to improve this limitation.^18^

Protein purification by affinity tagging and immobilization is a prominent technique in the field of biotechnology.^19^ Generally, this process involves the expression of recombinant proteins that incorporate a tag on either the N- or C-terminus. Immobilized metal affinity chromatography (IMAC) is a staple technique for protein purification. In these materials, a ligand is functionalized by chelation of a transition metal, such as nickel or copper. This immobilized metal has a strong affinity for a polyhistidine tag (typically composed of 6 consecutive histidine residues).^20^ This process allows for the specific separation of the target protein from other undesirable cellular constituents liberated by lysis.^21–23^

Previous work has shown the application of immobilized metal affinity substrates for ESI- and DESI-MS analysis of recombinant proteins and the monitoring of their enzymatic activity.^24^ The commercially available flat surface used in that study possesses a low binding capacity, not exceeding ∼1.661 femtomoles per mm^2^. Considered alongside the loss in sensitivity exhibited by DESI-MS when analyzing larger proteins,^10^ the shortcomings of using smooth, flat, functionalized surfaces with low binding capacity are evident. Little protein is recovered for subsequent analysis, and larger proteins cannot be detected at all, even as the reactions of these immobilized proteins can be monitored. Here, we demonstrate the use of alternative form factors of Ni-NTA substrates to improve protein detection sensitivity by increasing the specific surface area and binding capacity. This is achieved by incorporating commercially available Ni-NTA magnetic beads (magbeads) to sequester and purify protein from cell lysate, followed by DESI analysis. The high binding capacity of the beads (80 mg of protein per milliliter of settled beads) allows for the recovery of a greater amount of protein, while their close packing, once deposited over a flat surface, provides a much larger specific surface area than the flat surface on its own.

In addition to the benefits of efficient protein capture provided by their large binding capacity and impacts on desorption footprint-specific surface area, the magnetic nature of these beads allows for a streamlined analyte recovery process. Similar materials have been developed for the capture of organophosphates from milk^25^ and cannabinoids from human plasma.^26^ Later, this material was applied as the sampling substrate for magnetic particle spray mass spectrometry (MPS-MS) to detect various small molecule drugs.^27^ An important characteristic of MPS-MS was that the magnetic microtubules used for analyte capture were restricted from binding protein, thanks to a layer of bovine serum albumin (BSA), preventing protein interactions with the tubule core and interference with small molecule analysis. Other magnetic nanomaterials, known as bare iron oxide nanoparticles (BIONs), have been used to isolate recombinant protein A expressed directly from the culture of protein-secreting *E. coli*. This was accomplished thanks to the expression of the protein of interest with a terminal arginine-histidine tag. Following elution, the separation of protein A was confirmed by HPLC and SDS-PAGE.^28^ The use of magnetic nickel-decorated graphene nanomaterials (GP-Ni) in the separation of recombinant proteins has also been demonstrated by the capture of His-tagged sterol methyltransferase protein from a complex matrix, followed by elution with imidazole and confirmation by SDS-PAGE.^29^ The use of such novel adsorbents as BIONs and GP-Ni provides a significantly greater surface area for protein capture, susceptibility to magnetic fields for simplified recovery, and low costs when compared to other methods of protein separation.

Herein, we present a method for detecting His-tagged recombinant proteins using commercially available Ni-NTA beads as sampling substrates. These beads were used to capture recombinant S-adenosylhomocysteine nucleosidase (SAHN) and lactate oxidase (LOx) from *E. coli* lysate, after which the beads were analyzed by DESI-MS. The captured proteins retain their enzymatic activities while immobilized on the beads, as the conversion of their substrates to the appropriate products could be detected. Acidifying the desorption solvent disrupts the His-nickel interaction by protonating the histidine side chain, thereby allowing the release of the protein for direct analysis by DESI-MS.

## Experimental Methods

### Chemicals

LC-MS grade water, LC-MS grade methanol, sodium L-lactate, and ammonium acetate were purchased from Sigma-Aldrich (St. Louis, MO, USA). Thirty nanometer diameter Ni-NTA-functionalized magnetic beads (MagBeads) were obtained from Cube Biotech (Monheim am Rhein, Germany) as a 25% volume/volume slurry in 10% ethanol. Formic acid was obtained from EMD Chemicals (Gibbstown, NJ, USA).

Tryptone, yeast extract, potassium phosphate monobasic, potassium phosphate dibasic, sodium phosphate monobasic, sodium chloride, imidazole, and isopropyl β-D-1-thiogalactopyranoside (IPTG) were purchased from RPI (Mount Prospect, IL, USA). Glycerol was purchased from US Healthcare Products (New Haven, CT, USA). 4-(2-Hydroxyethyl)­piperazine-1-ethanesulfonic acid (HEPES) was acquired from Ambeed (Buffalo Grove, IL, USA).

### Mass Spectrometry

Experiments were carried out using a Linear Ion Trap Mass Spectrometer (LTQ XL, Thermo Scientific, Waltham, MA, USA) connected to a three-dimensional translational stage (Purdue University, West Lafayette, IN, USA) to move the sample surface at 20 μm/sec. An electrosonic spray ionization (ESSI) source was fabricated in-house using two coaxial fused silica capillaries, secured by a Swagelok T-piece.^30^ The inner capillary, for solvent delivery, had an inner diameter (I.D.) of 50 μm and an outer diameter (OD) of 220 μm. The outer capillary, for nitrogen sheath gas delivery, had an ID of 320 μm, an OD of 430 μm, and a length of 2 cm. The sprayer was positioned to have an incident angle of 54° while maintaining a 4 mm distance back from the extended ion transfer capillary and a height of 1 mm up from the sampling surface. Spray potential was applied to the needle of a stainless-steel syringe used to deliver solvent, through the solvent capillary, to the source tip.

For the positive mode analysis of His_6_-SAHN, the spray potential was set to 4 kV, while the capillary temperature was maintained at 290 °C. The tube lens voltage and ion transfer tube (ITT) voltage were set to 188 V and 40 V, respectively. For the small molecule analysis of SAHN enzymatic activity, the ITT temperature was lowered to 250 °C, while the tube lens voltage was lowered to 75 V and the capillary voltage was lowered to 20 V. For the negative mode analysis of His_6_-LOx, the absolute values for these settings were maintained, but the polarity was switched. In short, the spray voltage was −4 kV, the tube lens was set to −188 V, and the ITT voltage was set to −40 V. Likewise, for the analysis of enzymatic activity, the tube lens was set to −75 V, and the ITT was set to −20 V. The ITT temperature remained at 290 °C for protein analysis and 250 °C for the detection of enzymatic activity.

### Protein Expression

Recombinant S-adenosylhomocysteine nucleosidase (SAHN, 25.4 kDa, EC 3.2.2.9) was cloned into the pET29b vector to generate a C-terminally His-tagged protein. This plasmid was transferred into *E. coli* BL21 (DE3), and the strain was grown in 1 L of Luria broth (LB) with 50 mg/L kanamycin at 37 °C until an OD_600_ of approximately 0.6 was reached. The temperature was then decreased to 20 °C, and expression was induced with the addition of 100 μM isopropyl β-D-1-thiogalactopyranoside (IPTG) and cultured overnight. After harvesting the cells by centrifugation, the cells were lysed by sonication, and the lysate was cleared by further centrifugation. In some experiments, the His-tagged SAHN was purified from the cleared lysate at room temperature using a hand-packed 15 mL Ni-NTA resin column (G Bioscience, St. Louis, MO, USA) following the manufacturer’s protocol. The purified protein was stored at −80 °C until spiked into control clarified lysate to a known concentration of 14.3 μM.

Recombinant *Enterococcus durans* lactate oxidase (LOx, 40.7 kDa, EC 1.1.3.2) was expressed in *E. coli* BL21­(DE3) cells transformed with a pET29b vector encoding a C-terminal His-tagged construct. Cells were grown in PEM medium (12 g/L tryptone, 24 g/L yeast extract, 40 mL/L glycerol, 0.072 M K_2_HPO_4_, 0.017 M KH_2_PO_4_) at 37 °C with shaking until an OD_600_ of approximately 0.8 was reached. Cultures were then cooled to 20 °C, and protein expression was induced with 100 μM IPTG overnight. Cells were harvested by centrifugation and resuspended in lysis buffer containing 50 mM NaH_2_PO_4_, 300 mM NaCl, 20 mM imidazole, and 10% glycerol (pH 8.0). The cell suspension was lysed by sonication, and the lysate was clarified by centrifugation at 19,000 × g for 20 min at 4 °C. The resulting supernatant was applied to a Ni^2+^-affinity chromatography column (G-Biosciences, St. Louis, USA) pre-equilibrated with lysis buffer. After washing with lysis buffer to remove nonspecifically bound proteins, the target protein was eluted using buffer containing 250 mM imidazole. The eluted protein was concentrated and further purified by size-exclusion chromatography on a 16/600 Superdex pg column (Cytiva, Wilmington, DE, USA) using an FPLC system equilibrated in 40 mM HEPES, 100 mM NaCl, and 10% glycerol (pH 7.5).

The clarified lysate was prepared from *E. coli* BL21­(DE3) cells grown in PEM medium (12 g/L tryptone, 24 g/L yeast extract, 40 mL/L glycerol, 0.072 M K_2_HPO_4_, 0.017 M KH_2_PO_4_) at 37 °C with shaking overnight. Cells were harvested by centrifugation and resuspended in lysis buffer containing 50 mM NaH_2_PO_4_, 300 mM NaCl, 20 mM imidazole, and 10% glycerol (pH 8.0). The suspension was lysed by sonication, and the lysate was clarified by centrifugation at 19,000 × g for 20 min at 4 °C. The resulting supernatant was collected as the soluble lysate fraction.

### Protein Capture

Purified protein was spiked into the prepared cell lysate to provide a concentration of 14.3 μM His-SAHN or His-LOx. The samples were prepared to this protein concentration based on the maximum eGFP binding capacity to the magbeads, as estimated based on reporting by the manufacturer.^31^ 10 μL aliquots of bead slurry were added into 500 μL clarified lysate spiked with recombinant protein, and after the addition of the magbeads, the solution was shaken at room temperature for 1 h. The magbeads were then magnetically pelleted from the solution, after which the supernatant was discarded. This pellet was rinsed vigorously with 100 mM ammonium acetate. After rinsing, the pellet was reconstituted to the original 25% v/v in ammonium acetate, and 2 μL spots of this slurry were then prepared for DESI-MS analysis.

### Enzyme Activity Monitoring

Ten microliter of magbead slurry was added to 500 μL of standard lysate spiked with either His-SAHN or His-LOx. This solution was shaken for 1 h to allow protein capture. After recovery and thorough rinsing of the bead pellet with 100 mM ammonium acetate, the magbeads were reconstituted to their original volume and concentration (10 μL, 25% v/v) in 100 mM ammonium acetate. Two μL aliquots of the recovered protein-adsorbed bead slurry were deposited for DESI-MS analysis. After the protein-adsorbed magbead spots dried, 2 μL aliquots of the substrates, 500 μM SAH in 100 mM ammonium acetate (for His-SAHN) or 500 μM lactate in 100 mM ammonium acetate (for His-LOx), were deposited over top. These spots were allowed to incubate in a lab-constructed humidity chamber at room temperature for 1 h, after which the residual liquid solution was allowed to dry under vacuum prior to analysis. Negative control spots were prepared by adding 10 μL of the original bead slurry to 500 μL of lysate in the absence of spiked protein. These beads were treated as described above: shaken, rinsed, and reconstituted to their original volume and concentration. Two μL spots of the protein-free bead slurry were then prepared, and 2 μL of either substrate in 100 mM ammonium acetate solution was deposited over top.

### Data Analysis

Mass spectra were collected using Xcalibur software and analyzed in Qual Browser (Thermo Scientific, Waltham, MA, USA). MagTran was used to deconvolute protein mass spectra and obtain a zero-charge spectrum.^32^

## Results and Discussion

Figure S2A demonstrates that nonspecific binding to the beads is low and that no discernible protein spectra were observed following incubation of the beads in clarified cell lysate. The baseline can be further improved by the inclusion of a low concentration of imidazole in lysis buffers. Imidazole is included in lysis and loading buffers in conventional IMAC assays to prevent the nonspecific binding of other histidine-rich proteins.^33,34^ Doing so provides minimal impact on recombinant protein yield while ensuring greater specificity in capture for subsequent analysis. This is demonstrated in the comparison of the protein captured and analyzed using the described protocol, with and without 20 mM imidazole in the lysis buffer (see Figures S2B and S2C in Supporting Information). Similar peak intensities are observed whether imidazole is present or not. However, with imidazole present during protein binding, baseline noise is reduced and S/N increases. Imidazole is removed by the vigorous rinsing carried out after capture, avoiding any potential ionization suppression during DESI-MS analysis.

SAHN is an enzyme possessing a multitude of functions, acting to catalyze the hydrolysis of the glycosidic bonds of several adenosine-containing metabolites, such as 5′-methylthioadenosine (MTA) and S-adenosyl homocysteine (SAH), producing free adenosine and a ribose-containing product (Eq. 1).^35,36^


1

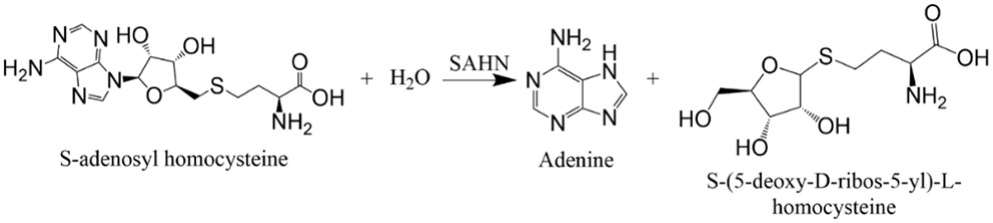




Recombinant His-SAHN was clearly detected by DESI-MS from dried spots of beads used to capture the protein from *E. coli* lysate. Clarified lysate containing His-SAHN was incubated with the Ni-NTA magbeads, after which the beads were pelleted, washed, and reconstituted to their original slurry volume in 100 mM ammonium acetate (Figure 1). To prevent their loss during the desorption process, the beads were deposited over top of a strip of two-sided adhesive tape stuck to a polished glass slide (shown in Figure S1). Magnetism (provided by the field of either N42 or N52 neodymium magnets) by itself was unfortunately of insufficient strength to withstand the force of the DESI emitter employed. Spot sizes of ∼1.77 mm^2^ were obtained, providing an estimated surface density of ∼2.0 × 10^13^ beads/mm^2^ and a protein concentration of ∼0.8 nmol/mm^2^. This number of beads provides a maximum surface area of ∼100 000 mm^2^ (calculations shown in Supporting Information) compared to the nominal spot size mentioned above. The inclusion of 0.2% formic acid (FA) brings the local pH below the p*K*_a_ of the histidine side chain, interrupting protein binding and facilitating release from the bead surface. Our previous work demonstrated the ability to detect His-SAHN from Ni-NTA well plates using FA-containing solvents for release, followed by direct infusion ESI of the collected elution fraction. However, detection of consecutive protein charge states directly from a flat Ni-NTA slide was not observed, as shown in Figure S3.^24^ Figure 2 shows that when the specific surface area and protein binding capacity are substantially increased with the use of commercial Ni-NTA magbeads, DESI-MS was able to clearly show the protein envelope of His-SAHN. The His-SAHN charge state envelope could easily be deconvoluted, and the zero-charge mass spectrum is shown in Figure S4.

**Figure 1:**
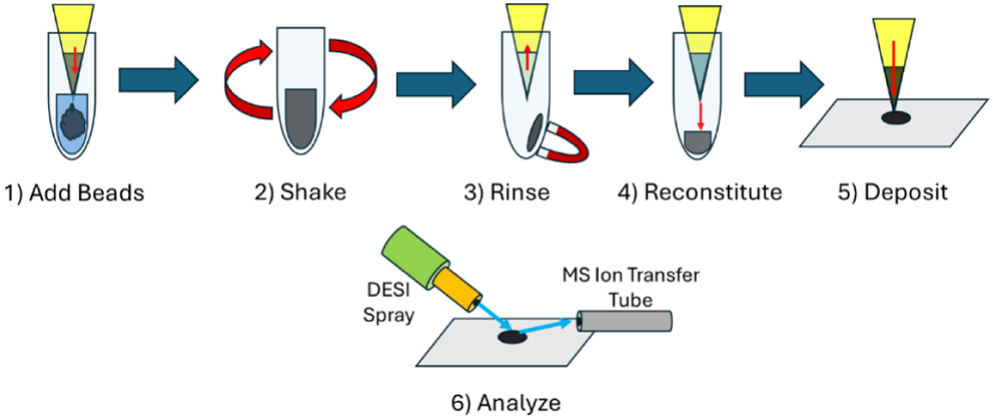
Schematic diagram of magbeads workflow for DESI-MS of recombinant proteins. 1) Beads are added to the cell lysate containing the recombinant protein. 2) The solution is shaken for 1 h. 3) Beads are magnetically pelleted, the supernatant is removed, and the pellet is repeatedly resuspended and washed. 4) The magbeads are reconstituted to the original bead concentration. 5) Protein-adsorbed magbeads are deposited on a glass slide. 6) MagBead spots are analyzed by DESI-MS.

**Figure 2:**
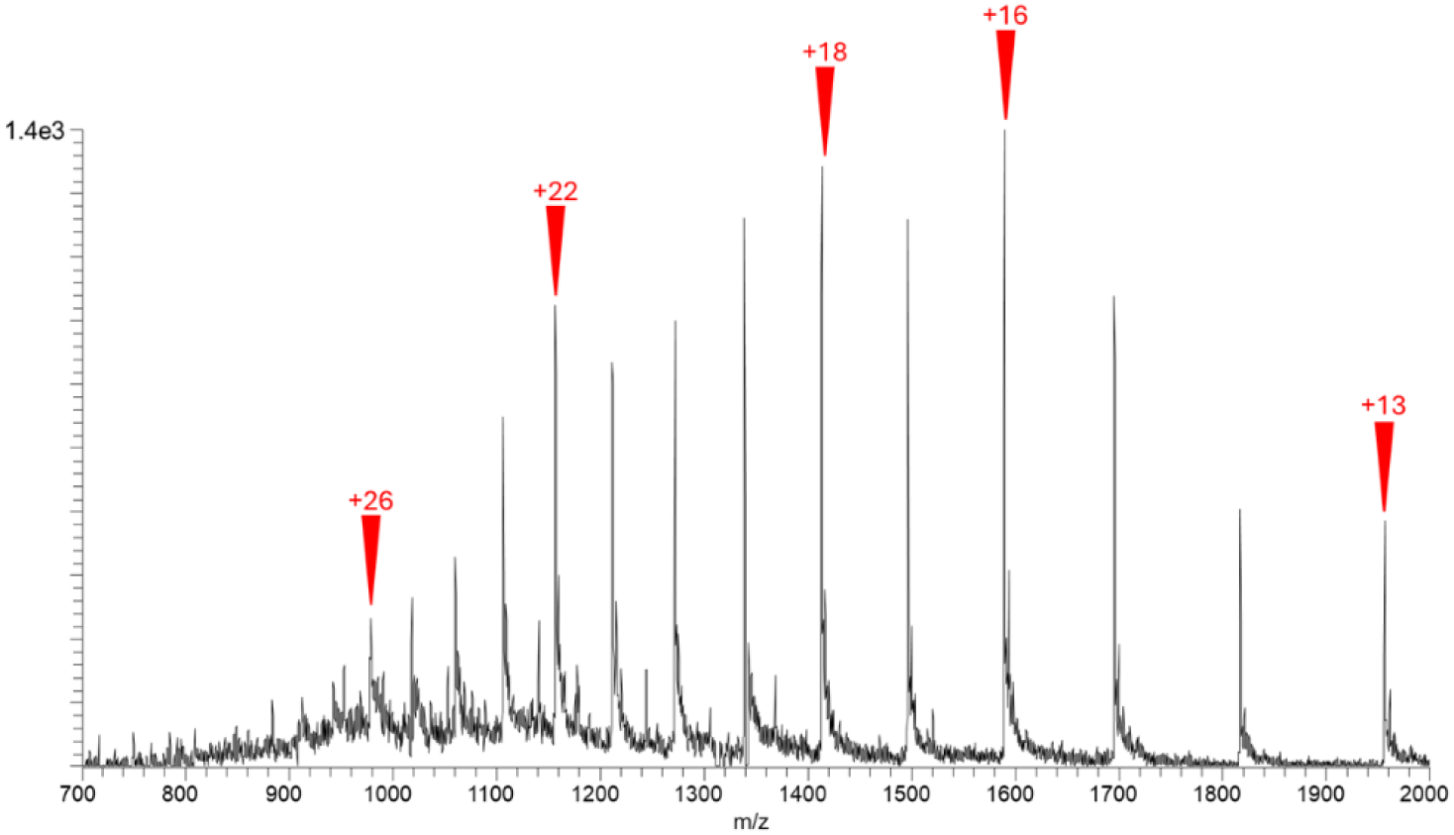
DESI-MS spectra of His-SAHN were collected from 2 μL of a 25% magbead solution deposited on an adhesive surface after protein capture from *E. coli* lysate containing 14.3 μM His-SAHN, ∼0.8 pmol/mm^2^ on surface.

The enzymatic activity of His-SAHN sequestered on beads was also observed after incubation with its substrate, S-adenosyl homocysteine. MagBeads were used to capture protein from clarified lysate and further prepared as described above. The deposited bead spots were then incubated with 2 μL drops of SAH in 100 mM ammonium acetate. After incubation in a humidity chamber for 1 h, we were able to detect the SAHN reaction products using a spray solvent of 80% methanol. Both products, adenine (*m*/*z* 136) and S-(5-deoxy-D-ribose-5-yl)-l-homocysteine (*m*/*z* 268), were observed in Figure 3B while absent from Figure 3A, the no-protein control, where the substrate S-adenosyl homocysteine appears at *m*/*z* 385. Sodium adducts (M+Na^+^) were also observed for both the substrate (*m*/*z* 407 in Figure 3A) and for one of the reaction products, S-(5-deoxy-D-ribose-5-yl)-l-homocysteine (*m*/*z* 290 in Figure 3B).

**Figure 3:**
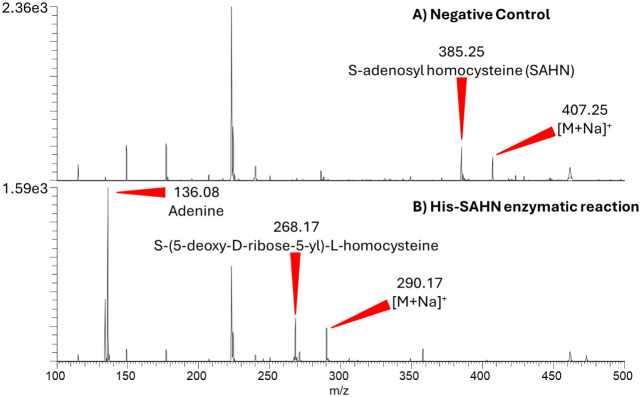
Enzymatic reaction of His-SAHN on MagBeads after capture from lysate. A) Negative control: 500 μM SAH in 100 mM ammonium acetate overtop of beads with no protein captured and B) His-SAHN reaction: 500 μM SAH in 100 mM ammonium acetate overtop of beads used to capture the His-SAHN protein.

Lactate Oxidase (LOx) is one of many enzymes featuring a flavin mononucleotide (FMN) cofactor that oxidize α-hydroxy acids. Specifically, in the presence of oxygen, this enzyme converts lactate to pyruvate and hydrogen peroxide (eq 2).^37–39^


2

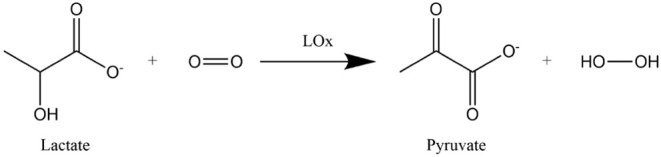




LOx has been used in biosensors for detecting lactate,^40^ an important metabolite to monitor in sports medicine, as its accumulation may hinder muscle performance through acidosis.^41^ The recombinant His-LOx was detected at a relatively low signal-to-noise ratio when subjected to DESI-MS analysis from the magbeads. This protein was treated in an identical manner as His-SAHN. The protein was captured from *E. coli* lysate with MagBeads, after which the beads were pelleted by a magnet and rinsed vigorously, followed by reconstitution to their original volume and concentration (10 μL and 25% v/v), and finally, deposition of 2 μL spots of bead slurry onto an adhesive. The same solvent system, including FA, was used for this analysis as well, facilitating the release of His-LOx from the bead surface, producing the spectrum shown in Figure 4. The deconvoluted, zero-charge mass spectrum for the LOx charge state distribution is shown in Figure S5.

**Figure 4:**
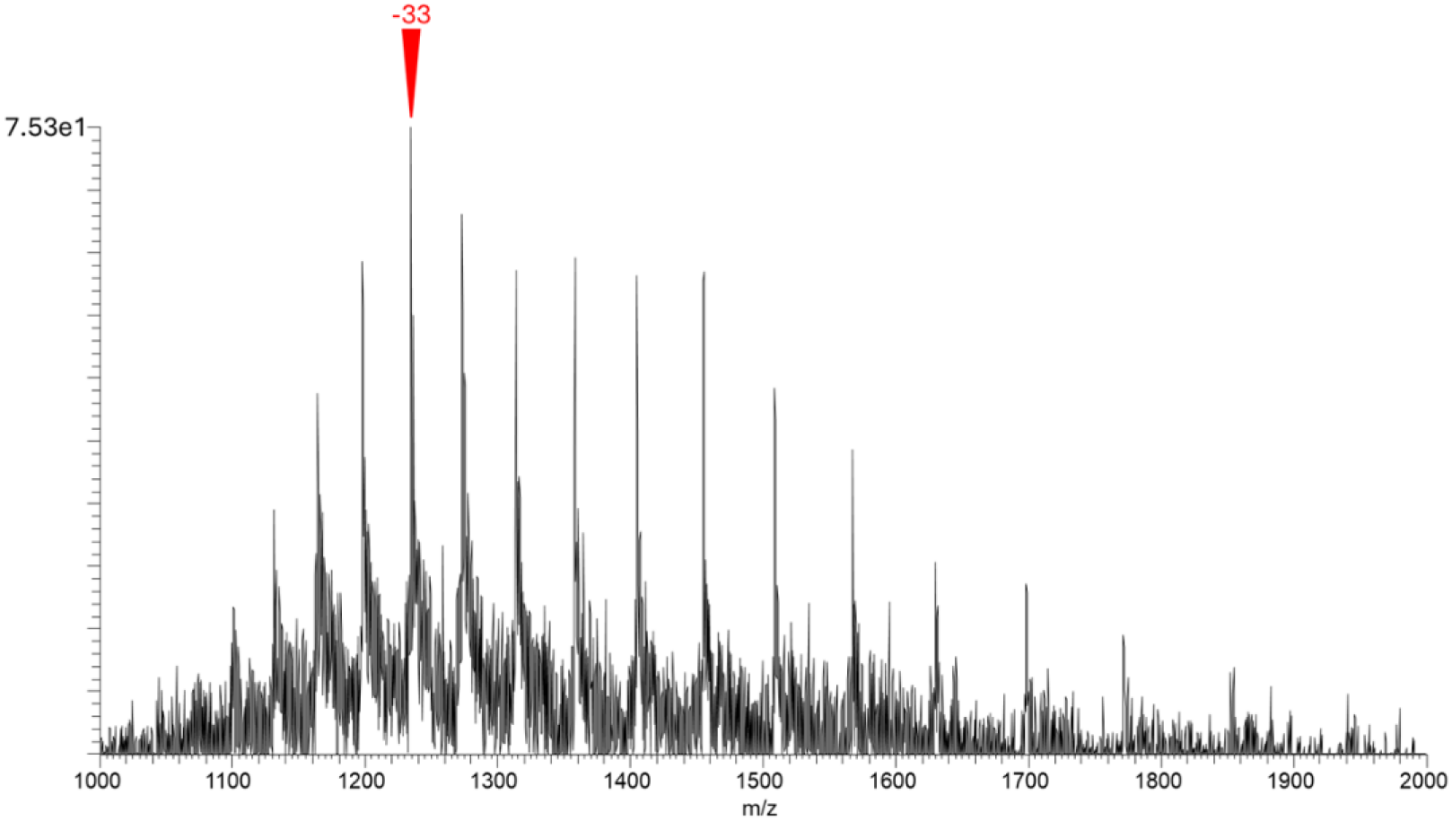
DESI-MS spectra of His-LOx collected from 2 μL of a 25% MagBead solution deposited on an adhesive surface after protein capture from *E. coli* lysate containing 14.3 μM His-Lox, ∼0.8 pmol/mm^2^ on surface.

Like His-SAHN, His-LOx retains its enzymatic activity while immobilized on the bead surface, as shown in Figure 5. Two μL drops of a substrate solution, 500 μM sodium lactate in 100 mM ammonium acetate, were deposited over dried bead spots used to capture the protein. Controls were prepared, as with His-SAHN, by depositing 2 μL drops of the substrate solution over dried 2 μL drops of the 25% bead slurry that had not been used to capture any His-LOx. In the control (Figure 5A), we observe only the lactate substrate peak at *m*/*z* 89. In the spectra collected from lactate incubated for 1 h with a spot of beads with immobilized protein (Figure 5B), we observe the depletion of *m*/*z* 89 and near-complete conversion to *m*/*z* 87, corresponding to pyruvate. Despite the near-total attenuation of *m*/*z* 89 in the reaction spectra, implying total conversion, the pyruvate peak detected in the reaction spectra is nearly half as intense as the lactate peak in the negative control. This difference in intensities can likely be explained by differences in ionization efficiencies. An equimolar concentration of the two analytes produced a spectrum where lactate produced ion intensities twice that of pyruvate, as shown in Figure S6.

**Figure 5:**
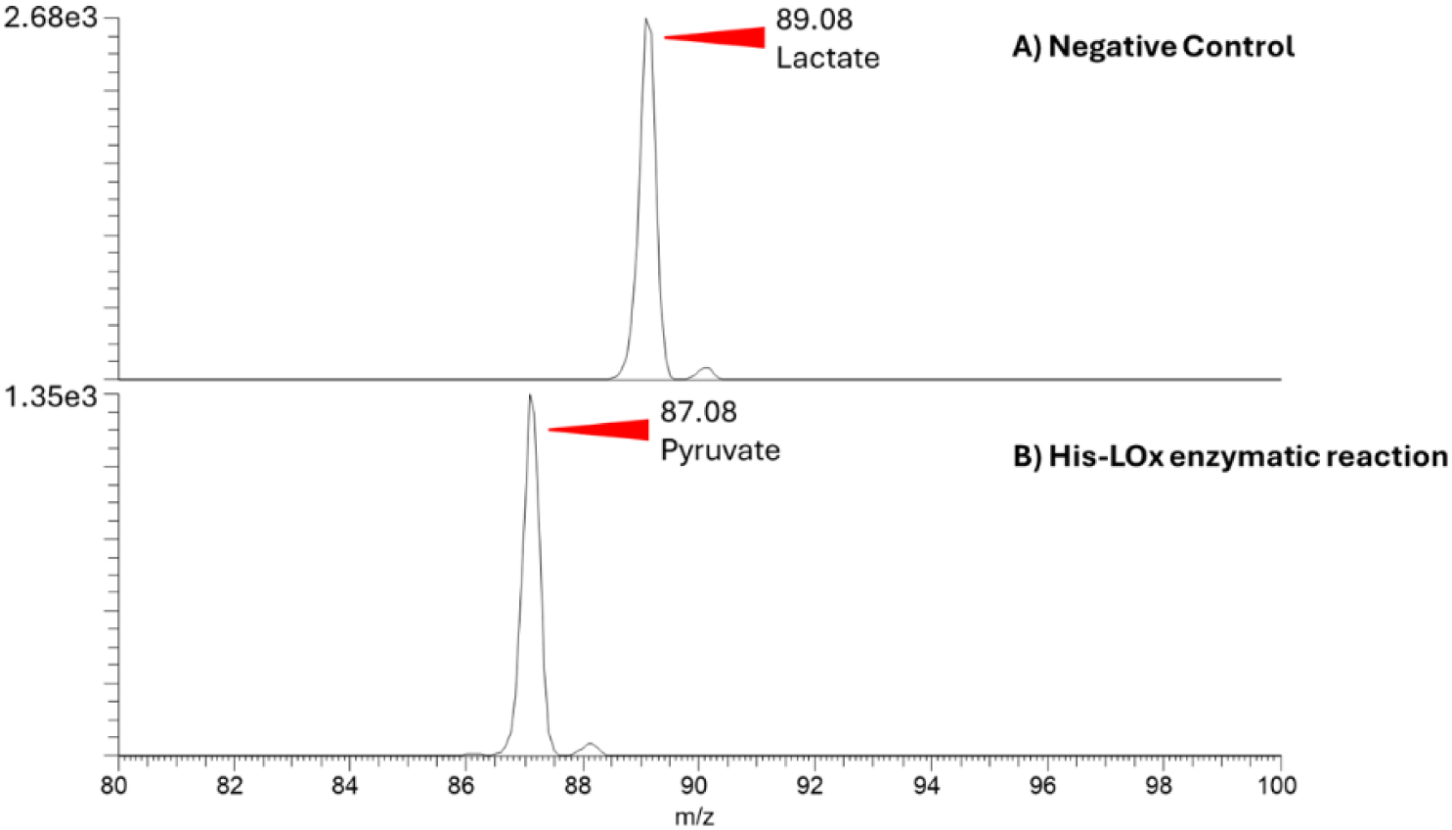
Enzymatic reaction of His-LOx on magbeads after capture from lysate. A) Negative control: 500 μM lactate in 100 mM ammonium acetate overtop of beads with no protein captured and B) His-LOx reaction: 500 μM lactate in 100 mM ammonium acetate overtop of beads used to capture the His-LOx protein.

The method demonstrated typical reproducibility for DESI-MS. For His-LOx, the response of the highest intensity charge state (HICS) possessed a relative standard deviation of ∼20%, while for His-SAHN, the response varied by 9.7%. Variability in the response for enzyme substrates and products was noticeably higher but can be further improved when using isotopically labeled standards. For the conversion of lactate to pyruvate by His-Lox, the standard deviation for the lactate control response was ∼24%, while the deviation in pyruvate response after the reaction was much higher at ∼42%. For the conversion of SAH to adenine and S-(5-deoxy-D-ribose-5-yl)-l-homocysteine, we observe a standard deviation of ∼38% for SAH in the control spectra, while the products exhibit deviations of ∼26% for adenine and ∼37% for S-(5-deoxy-D-ribose-5-yl)-l-homocysteine.

## Conclusions

In this work, we demonstrate the use of commercially available immobilized metal affinity beads (MagBeads) for the purification of recombinant proteins from *E. coli* cell lysate, followed by direct analysis with DESI-MS. Doing so allows us to carry out the analysis of enzyme expression fidelity and activity monitoring, and, in the future, to potentially deorphanize proteins with unknown functions, without the need for more costly and time-consuming methods of purification. By taking advantage of the increased binding capacity of the magbeads, alongside the increased specific surface area provided by their three-dimensional form factor, we are now able to easily detect proteins such as the 25.4 kDa His-SAHN and the 40.7 kDa His-LOx directly from *E. coli* lysate by DESI-MS. The magnetic nature of the beads enables a simple incubation and rinsing protocol prior to analysis, facilitating the method’s high-throughput protein characterization and identification. Because proteins retain activity when immobilized on the beads, these advantages extend to functional analysis as well. With these factors in mind, the inclusion of magnetic immobilized metal affinity beads in mass spectrometry workflows for protein characterization provides a notable improvement in efficiency. Considering the excellent spectral appearance for a 40.7 kDa protein using this protocol, we are hopeful that similar approaches may, in the future, be useful for extending the mass range of protein analysis by DESI-MS.

## Supplementary Material



## Notes

Photographs showing the immobilization of beads; spectra showing the role of imidazole during protein sequestering; spectra comparing the analysis of protein between glass slides and beads; deconvoluted spectra for the proteins; differences in ionization efficiency; and calculations of surface area and protein concentrations (PDF)
