# Supplementary material for: Immobilized Metal Affinity Beads for Recombinant Protein Analysis by Desorption Electrospray Ionization Mass Spectrometry
Source: J Am Soc Mass Spectrom. 2026 Jul 17;37(8):1964–70. doi: 10.1021/jasms.6c00155 (PMC13450393; doi:10.1021/jasms.6c00155)
Supplement: Supplementary file 1 [file js6c00155_si_001.pdf]

# Supporting Information

## Immobilized metal affinity beads for recombinant protein analysis by desorption electrospray ionization mass spectrometry

Christopher James Taylor, Sumana Parvin, Fazeleh Shafiepour, Frederick Stull, Andre R Venter*

*Department of Chemistry, Western Michigan University, Kalamazoo, Michigan, 49008-5413*

*Corresponding Author: andre.venter@wmich.edu

**Tables of Contents:**

Figure S1: Immobilization of Bead Spots

Figure S2: His-LOx spectra with and without imidazole.

Figure S3: Comparing the analysis of SHAN by DESI-MS from Ni-NTA glass slides and immobilized beads.

Figure S4: Zero-Charge Mass Spectra of His-SAHN.

Figure S5: Zero-Charge Mass Spectra of His-LOx.

Figure S6: Ionization efficiency investigation of lactate and pyruvate.

Supplemental Calculations

**Figures:**

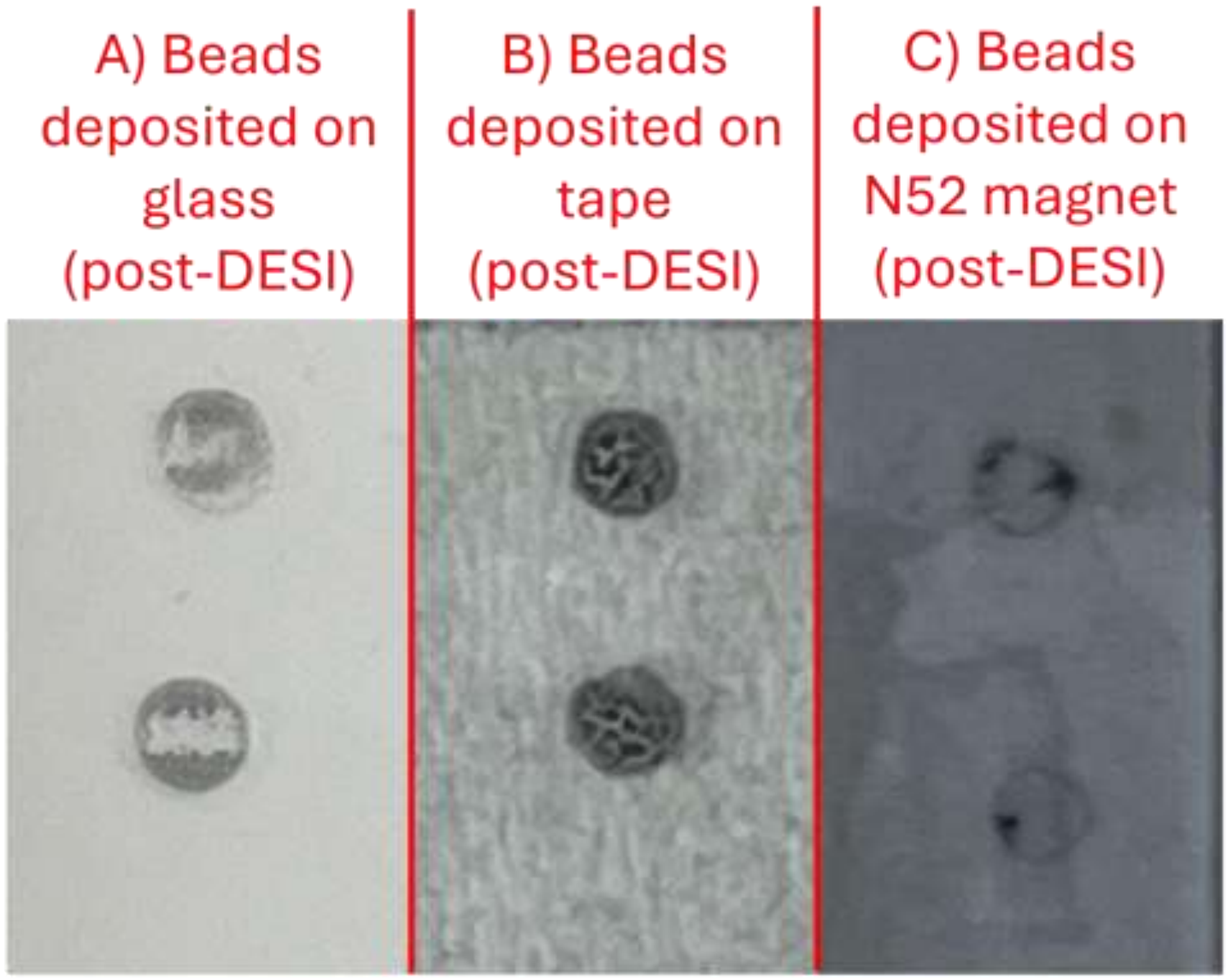


Figure S1. Beads spot A) on glass, B) on two-sided adhesive tape, and C) on an N52 magnet after subjection to desorption spray.

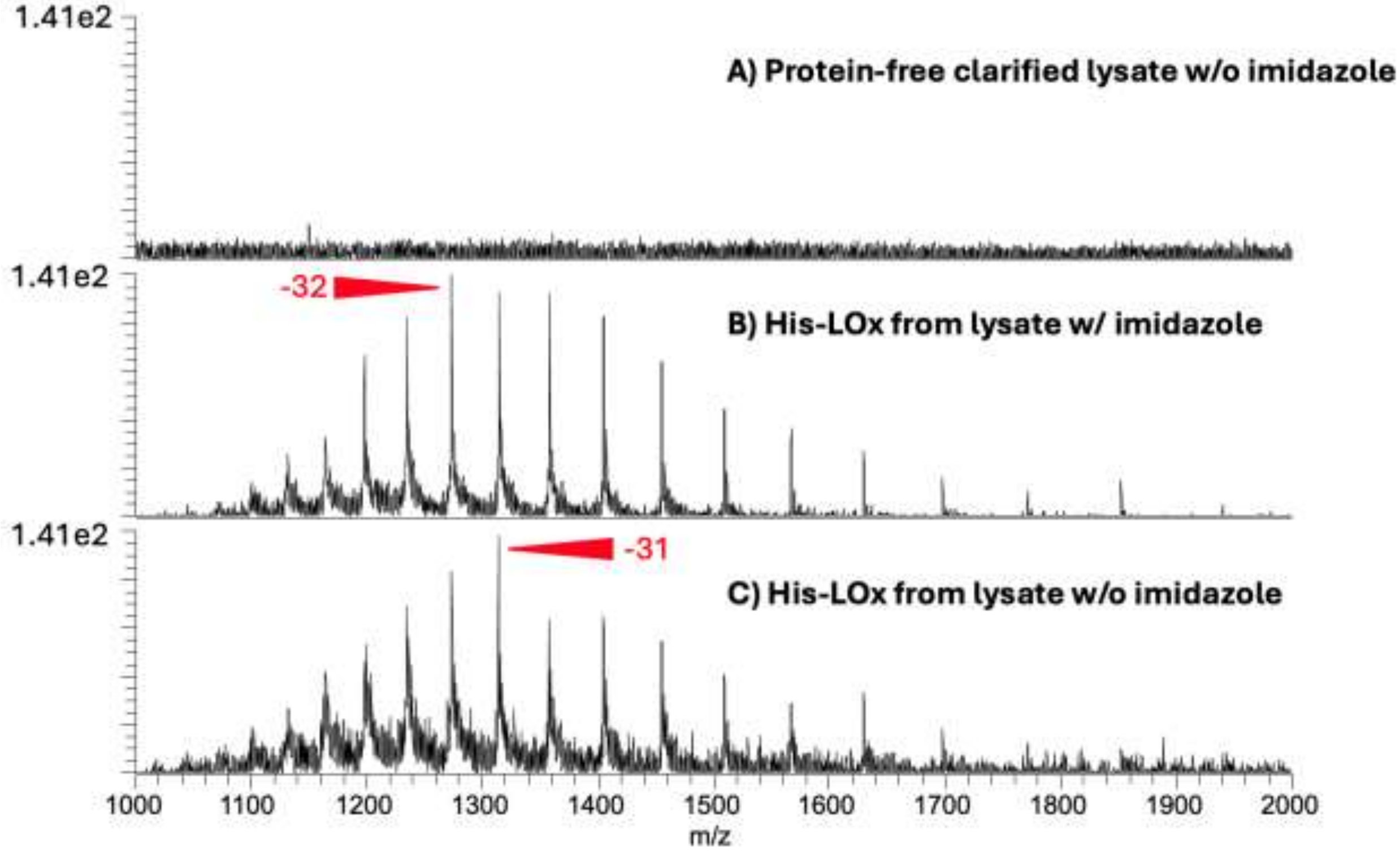


Figure S2: Mass spectra of A) protein-free clarified lysate without imidazole, B) His-LOx without imidazole included in lysis buffer, and C) with imidazole in lysis buffer.

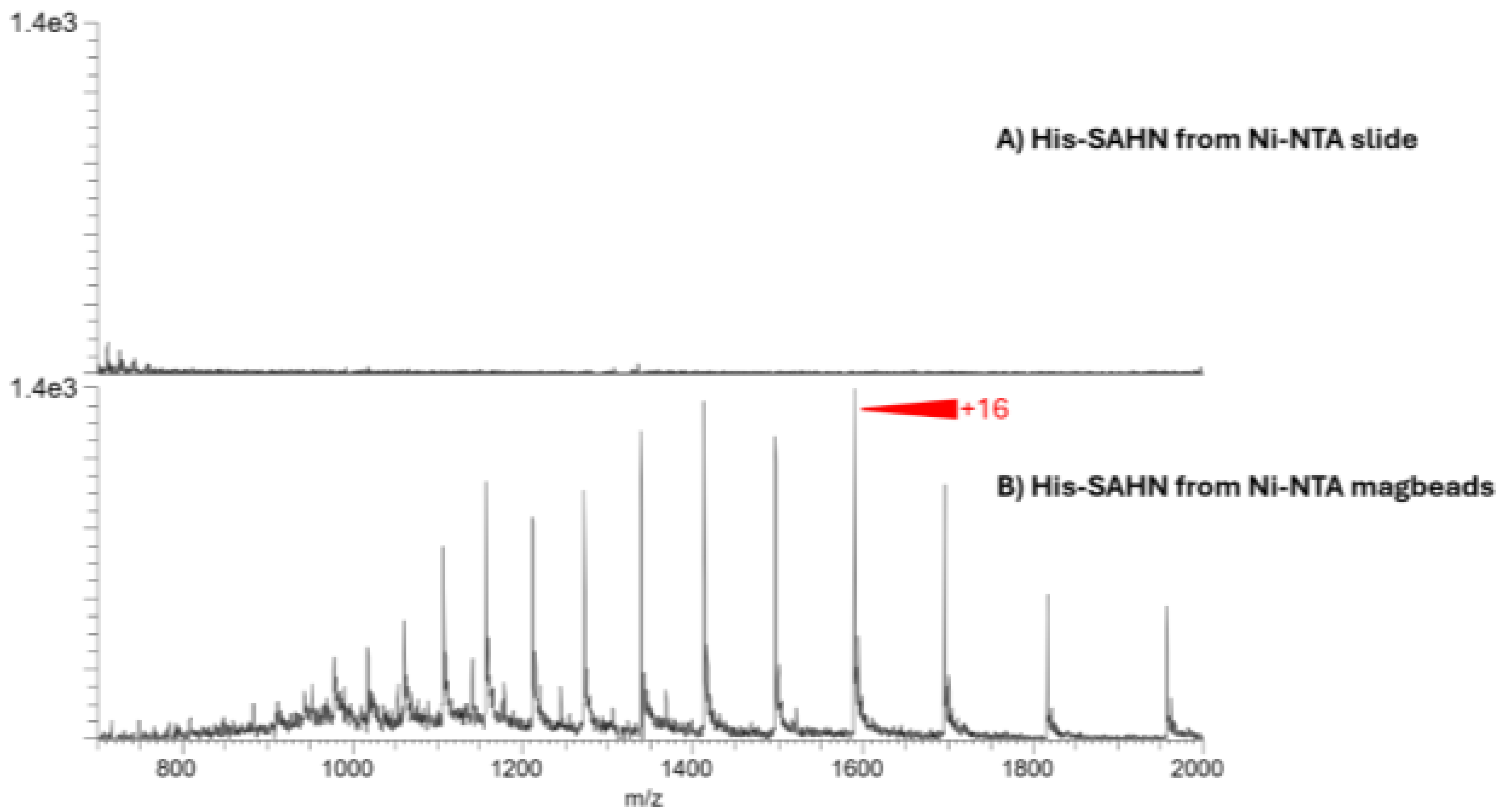


Figure S3. Comparison of DESI-MS spectra for His-SAHN from A) a Ni-NTA functionalized glass slide and B) from Ni-NTA magnetic beads immobilized on a glass slide.

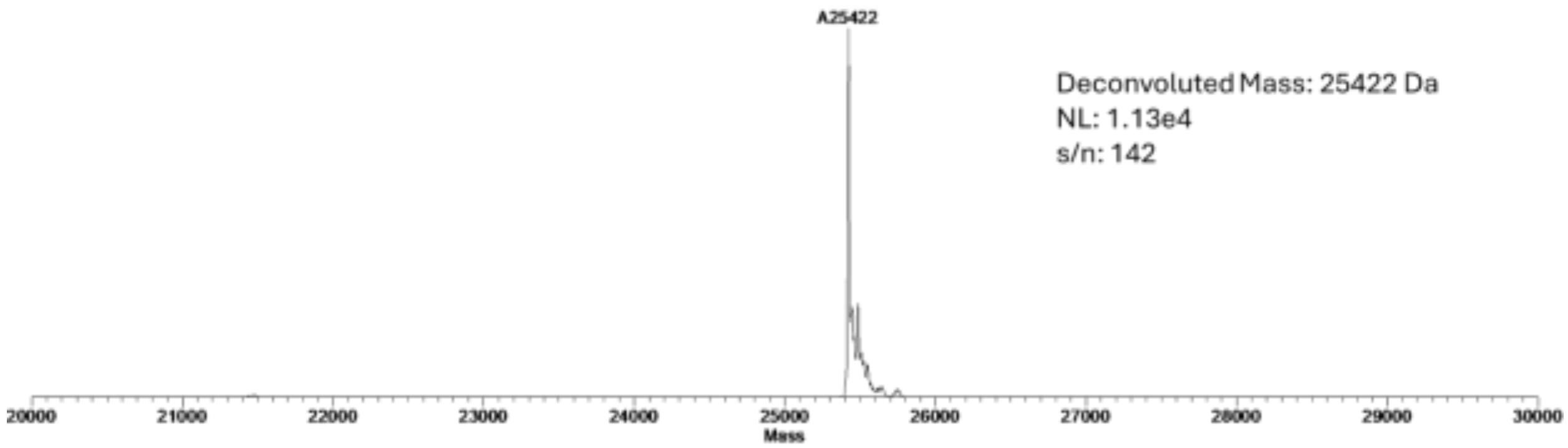


Figure S4. Zero-charge mass spectra of His-SHAN from magbeads.

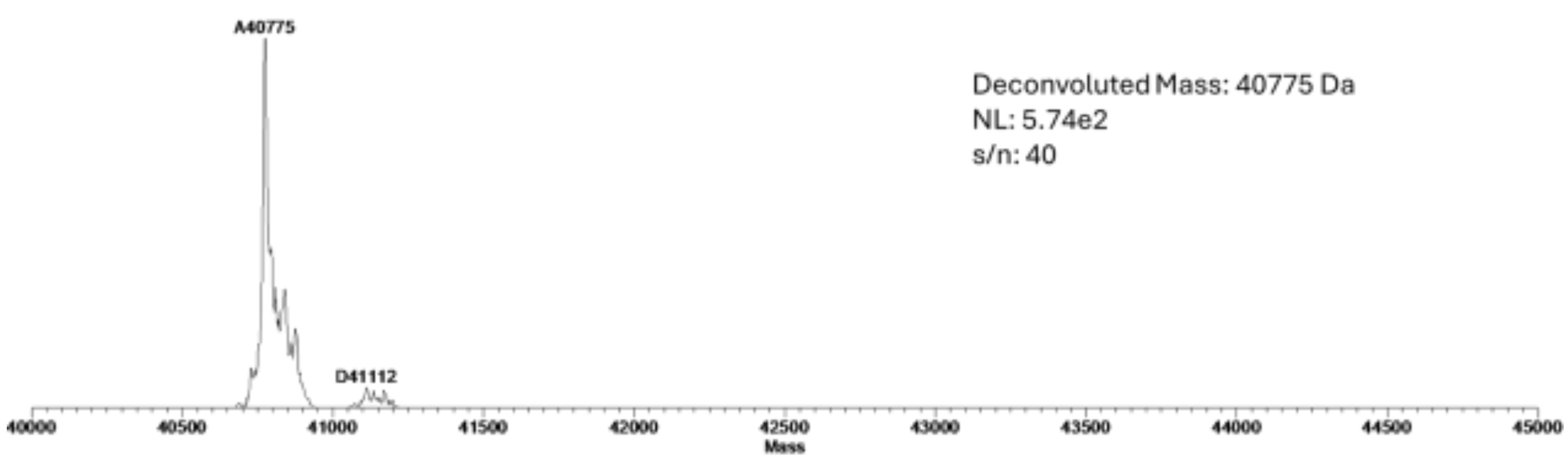


Figure S5. Zero-charge mass spectra of His-LOx

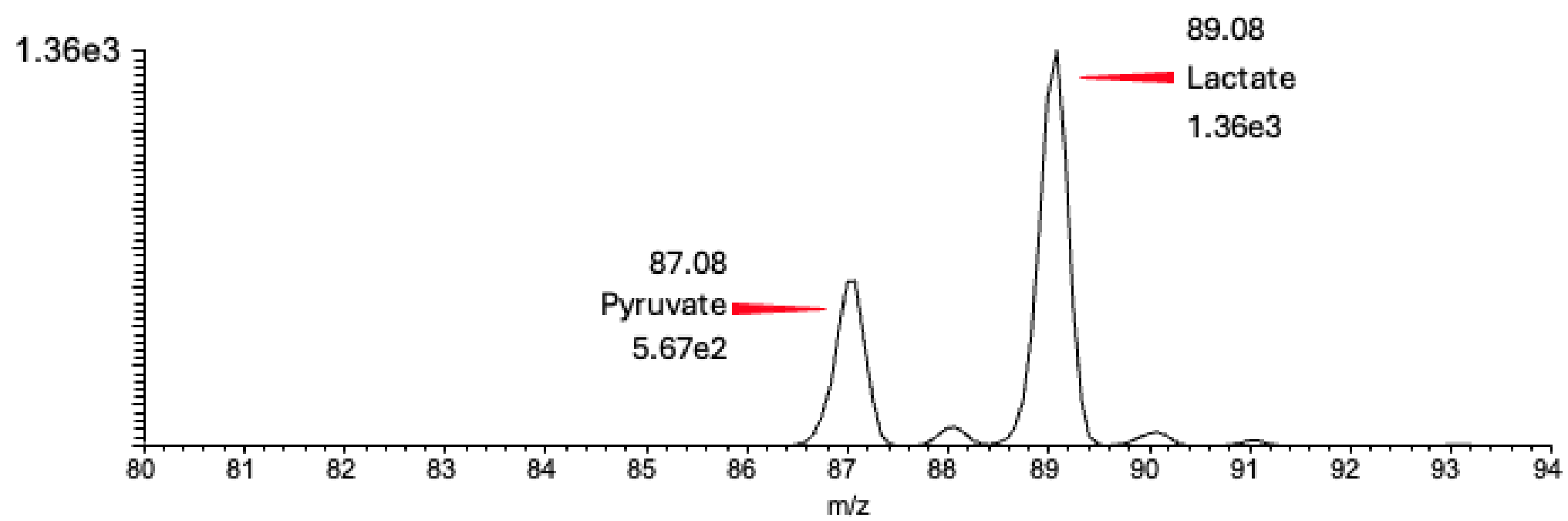


Figure S6: Ionization efficiency investigation of lactate and pyruvate. Direct ESI-MS spectra of 5 µM lactate and pyruvate in 80% methanol analyzed on LTQ XL.

**Supplemental calculations/ estimates**

Surface density of bead spots (beads per spot/ drop and beads per unit area):

A 2 µL drop of the 25% v/v slurry contains 0.5 µL worth of beads

A single 30 nm diameter bead provides a volume of 14134 $nm^3$ ($1.4134 \times 10^{-14}$ µL)

$$Vol = \left(\frac{4}{3}\right)\pi r^3 = \left(\frac{4}{3}\right)\pi\left(\frac{30\ nm}{2}\right)^3 = 14134\ nm^3\ per\ bead = 1.4134 \times 10^{-14} \mu L\ per\ bead$$

This would mean that roughly $3.538 \times 10^{13}$ beads could fit into 0.5 µL bead bed (assuming 25% slurry composition reported by the manufacturer considers spherical packing) and thus the 2 µL slurry droplet.

$$Beads\ in\ 2\ \mu L\ droplet = \frac{(volume\ of\ bead\ fraction)}{(bead\ volume)} = \frac{0.5\ \mu L}{1.4134 \times 10^{-14}\ \frac{\mu L}{bead}}$$

$$= 3.538 \times 10^{13}\ beads\ in\ 2\ \mu L\ droplet$$

Given an average surface area per spot of ~1.77 $mm^2$, this would provide a surface density of ~$2.0 \times 10^{13}$ beads per $mm^2$ of covered surface.

$$\frac{3.538 \times 10^{13}\ beads}{1.77\ mm^2} = 1.999 \times 10^{13}\ beads\ per\ mm^2 = \sim 2.0 \times 10^{13}\ beads\ per\ mm^2$$

Maximum surface area provided by beads

The maximum area provided by the bead spots can be estimated by finding the surface area of each bead and multiplying by the total number of beads per 2 µL spot

maximum area=(bead area) × (number of beads deposited)=($4\pi r^2$ )(3.538 1× $10^{13}$ )

$$maximum\ area = 4\pi \left( \frac{3 \times 10^{-5}\ mm}{2} \right)^2 \times\ (3.538X10^{13})$$

$$= 2.827X10^{-9}\ \frac{mm^2}{bead} \times\ (3.538X10^{13}\ beads) = 100034.59\ mm^2$$

$$=\ \sim 100000\ mm^2$$

<u>Protein surface concentration over area of bead spot coverage</u>

A 500 µL solution of 14.3 µM total protein should contain 7.15 × $10^{-3}$ µmol

$$5X10^{-4}\ L \times \frac{14.3\ \mu mol}{L} = 7.15 \times 10^{-3}\ \mu mol$$

Assuming complete recovery onto the 10 µL bead slurry.

2 µL protein-containing slurry is deposited onto the surface, providing 1.43 × $10^{-3}$ µmol protein

Divided by the average surface area (1.77 $mm^2$), this provides the total amount of protein per unit area of deposited bead slurry after protein capture, which is ~0.800 nmol/$mm^2$

$$\frac{1.43 \times 10^{-3} \mu mol}{1.77\ mm^2} = 0.800\ \frac{nmol}{mm^2}$$
